# Color change of the bleached enamel treated with calcium silicate- sodium phosphate-sodium monofluorophosphate-based system

**DOI:** 10.4317/jced.55286

**Published:** 2019-04-01

**Authors:** Gisele Carneiro, Débora Monteiro, Marcela Rodrigues, Monica Yamauti, Allyson Moreira, Cláudia Magalhães

**Affiliations:** 1 DDS, Department of Restorative Dentistry. School of Dentistry, Universidade Federal de Minas Gerais; 2 DDS, MSc, PhD student, Department of Restorative Dentistry. School of Dentistry, Universidade Federal de Minas Gerais and Professor, Faculdade de Estudos Administrativos, Dentistry, FEAD; 3 DDS, MSc, Department of Restorative Dentistry. School of Dentistry, Universidade Federal de Minas Gerais; 4 DDS, MSc, PhD, Professor, Department of Restorative Dentistry. School of Dentistry, Universidade Federal de Minas Gerais; 5 DDS, PhD, Professor, Department of Restorative Dentistry. School of Dentistry, Universidade Federal de Minas Gerais; 6 DDS, MSc, PhD, Professor, Department of Restorative Dentistry. School of Dentistry, Universidade Federal de Minas Gerais

## Abstract

**Background:**

The increasing demand of tooth bleaching has also increased the need of researches focusing on the durability of the resultant color. The aim of this study was to evaluate the effect of a system based on calcium silicate, sodium phosphate and sodium monofluorophosphate (Regenerate™) on color maintenance of bleached enamel considering two waiting times for the contact with a cola drink.

**Material and Methods:**

This *in vitro*study was performed on bovine enamel specimens (n = 100), bleached with 35% H2O2 and treated with: G1 Distilled water; G2 Artificial saliva; G3 RegenerateTM Serum and Toothpaste; G4 RegenerateTM Toothpaste; G5 RegenerateTM Serum. The groups G3, G4 and G5 received one application of the respective products for 3 min, in 3 consecutive days. The color parameters (ΔE, L*, a*, b*) were evaluated by spectrophotometry before and after bleaching and after surface treatments and immersion (15min) in cola drink, on the waiting times of 24 hours (T1) and 7 days (T2). The effect of surface treatments and waiting times was evaluated by Two-Way ANOVA, Kruskal-Wallis, Wilcoxon and T test (*p*<0.05).

**Results:**

There was no significant effect of the surface treatments (*p*=0.57), waiting times (*p*=0.97) and their interaction (*p*=0.47) considering ΔE. The analysis of repeated measures of the color coordinates L*, a* and b* showed a decrease of chromaticity (a*, b*) for G3, G4 and G5 and an increase of lightness (L*) for G5, after immersion in cola drink, suggesting some protection against bleached enamel pigmentation.

**Conclusions:**

RegenerateTM has a potential protective effect on bleached enamel color maintenance. The waiting times of 24 hours and 7 days for the contact with the cola drink did not influence bleached enamel color maintenance.

** Key words:**Tooth bleaching, hydrogen peroxide, spectrophotometry, pigmentation.

## Introduction

Despite the large number of studies regarding the effectiveness of several techniques for dental bleaching and their adverse effects, there are no consistent findings about its durability. The concerns are mainly related to how long patients should wait until consumption of staining foods and beverages (1,2).

In addition, another concern is the adverse effect caused by the bleaching agent on the enamel (3). It is known that bleaching agents with low pH values can lead to enamel chemical composition alteration, microhardness decrease, and morphological alteration (4,5). Scanning electron microscopy revealed a decrease on the enamel density after bleaching, concluding that the enamel demineralization and consequent corrosion may be caused by the bleaching agent (3).

Due to corrosion, the bleached enamel becomes more susceptible to pigmentation than the unbleached (6,7), which could reduce the durability of the resultant color. Considering the mineral loss induced by the whitening agents, researches have been investigating post-bleaching therapies to reduce the adverse effects of bleaching (8). The technology Regenerate Enamel Science™ was developed based on the combination of calcium silicate, sodium phosphate and fluoride salts and proposes to increase the enamel mineralization providing additional calcium and phosphate, which leads to the hydroxyapatite formation and consequent remineralization of hard dental tissues (9,10).

The aim of this study is to evaluate the effects of surface treatments with a system based on calcium silicate, sodium phosphate and fluoride considering the waiting times of 24h and 7 days until the contact with a cola drink on the color change of the enamel bleached with 35% hydrogen peroxide. The null hypothesis is that there is no difference between the surface tratments with Regenerate Enamel Science™ and waiting times of 24h and 7 days on the color change of bleached enamel.

## Material and Methods

This *in vitro* study was a randomized complete block design. The independent variables were the surface treatments (5 levels) and the waiting times until the contact with cola drink (2 levels). The dependent variable was the enamel color change evaluated with a spectrophotometer and expressed by the parameters ΔE, L*, a* and b*. The experimental specimens were 100 bovine incisors, randomly distributed in 10 complete blocks (n=10).

Data from a pilot study (n=4) were used to estimate the minimum sample size according the following parameters:

Mean ΔE of the Control Group = 9.0 

Mean ΔE of the Treatment Group = 14.35

Standard deviation of the variable = 3.9 

Standardized magnitude of effect = (14.35 - 9.0 / 3.96) = 1.352

For bilateral α = 0.05 and β = 0.80, the simplified formula for Student T-test was applied: N=16/(Standardized Magnitude of effect)2=16/1.352 =8.8

The minimum sample size estimated was 9 bovine teeth. To compensate possible losses it was added 10%, resulting in 10 teeth per group.

One hundred bovine incisors were extracted and stored in 0.1% thymol solution. They were sectioned on the cementum-enamel junction to remove the root and the crowns were cleaned with an ultrasound device and polished with pumice and water using a Robinson brush.

The pulps were extracted under irrigation with 2.5% sodium hypochlorite solution to remove the remaining organic tissues. The pulp chamber entrance was sealed with zinc oxide-eugenol paste (Lysanda Produtos Odontológicos, São Paulo, Brazil). The crowns were examined with an optical microscope (magnification = 8x) to exclude the specimens with surface defects. A 36mm2 area was delimited with an adhesive tape on the middle third of each buccal surface and two layers of nail polish (Risqué Niasi S.A., Taboão da Serra, Brazil) were applied. The adhesive tape was removed to expose the experimental area. On the palatal face, the specimens were identified (Pilot Pen do Brasil S/A, São Paulo, Brazil). The specimens were stored in distilled water under refrigeration at 8 ± 2°C for 96 hours until the beginning of the experiment. A spreadsheet for randomization in each block was generated using Microsoft Excel (Microsoft Corporation, Redmond, USA) and was applied.

The teeth were dried with an absorbent paper and stored in a dark box to avoid the interference of light. Then, the first evaluation of color was performed with a digital spectrophotometer (Vita EasyShade, Vita, Bad Sackingen, Germany). The color evaluations were registered according to the CIEL*a*b* tridimensional system. The color coordinates are: L*, which is achromatic and corresponds to lightness, ranging from 0 (black) to 100 (white); a*, corresponding to the green-red coordinate, being -a* green and +a* red; b*, which refers to the blue-yellow axis, since -b* is blue, and +b* is yellow. Three evaluations of L*, a* and b* were performed for each sample, and the mean was calculated.

All specimens were bleached with 35% hydrogen peroxide (Whiteness HP, FGM, Joinville, Brazil). Three applications were performed (15 minutes each), simulating one bleaching session without the application of light. After the three applications, the specimens were submitted to the second evaluation of color.

The specimens were distributed in five groups according to the randomized complete block design: G1- negative control, the specimens were immersed in distilled water; G2- positive control, the specimens were immersed in artificial saliva maintained in an incubator at 37 ± 1°C; G3- the specimens were submitted to a three minute application of Regenerate Enamel ScienceTM toothpaste and dual-phase calcium silicate/sodium phosphate serum for three consecutive days; G4- the specimens were submitted to a three minute application of the toothpaste for three consecutive days; G5- the specimens were submitted to a three minute application of the dual-phase serum for three consecutive days.

After the surface treatments, the specimens of group 1 were kept in distilled water (control) and the specimens of the other groups were maintained in artificial saliva in an incubator at 37 ± 1°C, considering the waiting times for the contact with the dye of 24 hours or 7 days.

Considering the respective waiting times of 24 hours or 7 days after the surface treatments, all specimens were immersed in 25 ml of cola drink (Coca-Cola, Rio de Janeiro, Brazil), for 15 minutes in an incubator at 37 ± 1°C. Then, the specimens were washed in distilled water, dried with an absorbent paper and the third color evaluation was performed.

**Figure 1:**
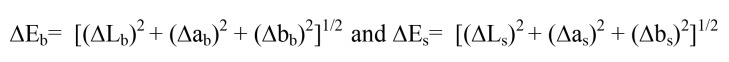
Formula.

The differences of L* (ΔL), a* (Δa) and b* (Δb) were calculated using the values obtained with the baseline evaluation, after bleaching and after staining. The color parameters ΔLb, Δab and Δbb were obtained by the difference between baseline and after bleaching values; and the color parameters ΔLs, Δas and Δbs were obtained by the difference between after bleaching and after staining values. To calculate the total difference of color obtained after bleaching (ΔEb) and after staining (ΔEs), the following formula was applied (1,11-13), (Fig. 1):

The value of ΔEb corresponds to the difference of color obtained with bleaching, while the ΔEs is the color change resulting from the pigmentation by the cola drink after the surface treatments. The smaller the ΔE, the smaller the color difference among the phases evaluated. According to CIEL*a*b* (1976), ΔE=1 is the smallest difference of color perceived by a device and ΔE≤3 is considered an acceptable color change (2). Some authors consider that from ΔE>3.3 (2) or ΔE>3.7 (12) the difference of color becomes unacceptable.

The statistical analysis was performed according to the randomized complete block design. Normal distribution and homogeneity of variance of the data were analyzed with Kolmogorov Smirnov and Levene tests. The effects of the surface treatments and the waiting times until the contact with the dye were evaluated by two-way ANOVA. The data L*0, b*0, a*0, L*b, b*b, a*b, L*s and b*s were evaluated by ANOVA and post-hoc Tukey’s Test, and a*s coordinate was evaluated by Kruskal-Wallis test.To assess the difference between the initial data, after bleaching and after pigmentation in each treatment group, Wilcoxon (a*s) and Student T test (L*0 a*0, b*0 / L*b, a*b, b*b / L*s, b*s) were applied. The confidence level applied was 5%. The statistical software SPSS 20 (Statistical Product and Service Solu¬tions, SPSS, Chicago, USA) was used for all tests.

## Results

-Analysis of the total color change (ΔE)

The median and interquartile distance for the color change data of all specimens after bleaching was ΔEb = 2.46 (1.51).

Data of the color change obtained after immersion in a cola drink (ΔEs), for the different surface treatment groups and different waiting times are shown in  Table 1. Two-way ANOVA showed that there was no significant effect of surface treatments (*p* = 0.575), waiting times (*p* = 0.977) and their interaction (*p* = 0.471) in the ΔEs values.

**Table 1:**
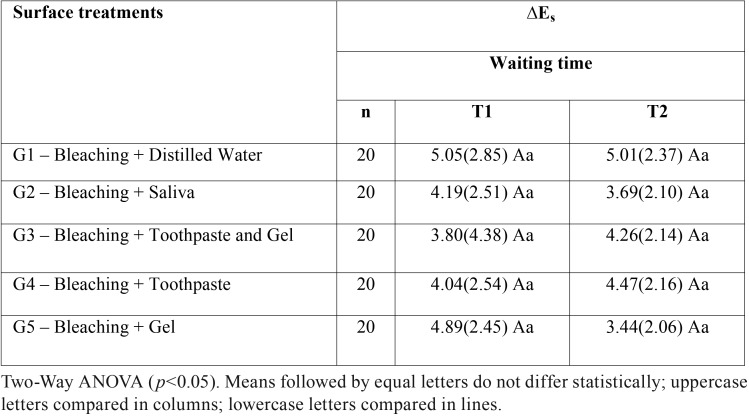
Mean (standard deviation) of the color change of the bleached enamel submitted to different surface treatments and contact with a cola drink after waiting times of 24 hours (T1) and 7 days (T2).

-Analysis of the color parameter L * (lightness axis)

There was no significant effect of the surface treatments at baseline L*0 (*p* = 0.707) and after bleaching L*b (*p* = 0.731). Student T test for repeated measures showed that the difference between L*0 and L*b was significant in groups G2 (*p*≤0.001), G3 (*p* = 0.033), G4 (*p* = 0.019) and G5 (*p* = 0.043), while in G1 (*p* = 0.165) there was no difference between the two evaluation moments. These results are presented in  Table 2.

**Table 2:**
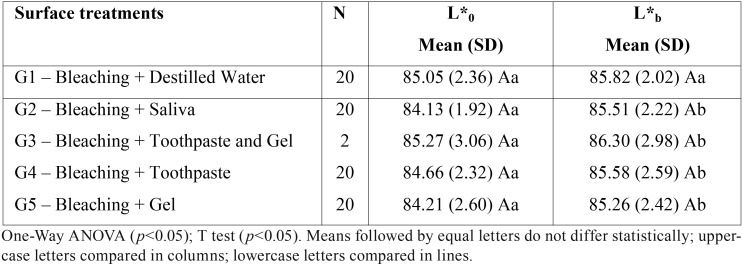
Mean (standard deviation) of the values of the L* coordinate (lightness axis), at baseline (L*0) and after bleaching (L*b), of the different surface treatments.

After immersion in cola drink (L*s), two-way ANOVA showed that there was no significant effect of surface treatments (*p* = 0.249), waiting times (*p* = 0.643), as well as their interaction (*p* = 0.056). Student T test showed that the difference between L*b and L*s was not statistically significant for G1 (*p* = 0.074), G2 (*p* = 0.102), G3 (*p* = 0.454), G4 (*p*= 0.146) and G5 (*p* = 0.164). These results are presented in  Table 3.

**Table 3:**
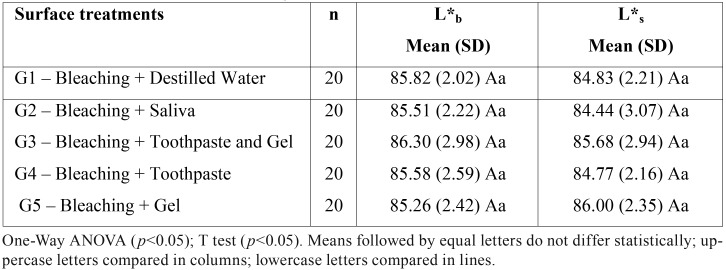
Mean (standard deviation) of the values of the L* coordinate (lightness axis) after bleaching (L*b) and after the contact with a cola drink (L*s) for the different surface treatments.

-Analysis of the color parameter a * (green-red axis)

Two-way ANOVA did not show a significant effect of the treatment groups at baseline a*0 (*p* = 0.447) and after bleaching a*b (*p* = 0.404). Student T-test revealed no difference between a*0 and a*b for groups G1 (*p* = 0.639), G2 (*p* = 0.846), G3 (*p* = 0,439), G4 (*p* = 0.836) and G5 (*p* = 0.945).

Kruskal-Wallis test did not reveal significant difference between groups after immersion in cola drink (a*s) (*p* = 0.312). Mann-Whitney test did not show a significant difference between the waiting times (*p* = 0.72) evaluated. The Wilcoxon test showed that the difference between a*b and a*s in each group was significant only for G2 (*p* = 0.046) and G5 (*p* = 0.018), whereas for G1 (*p* = 0.837), G3 (*p* = 0.94) and G4 (*p* = 0.588) there was no difference. The results are shown in  Table 4.

**Table 4:**
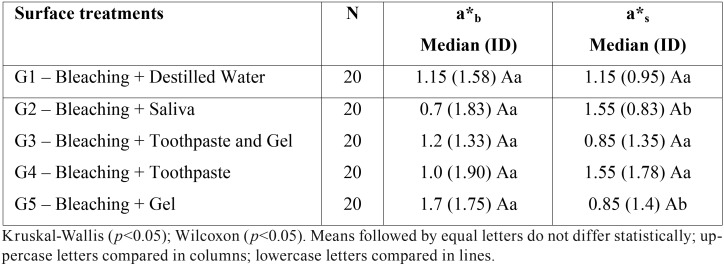
Median (interquartile distance) of the values of the a* coordinate (green-red axis) after bleaching (a*b) and after the contact with a cola drink (a*s) for the different surface treatments.

-Analysis of the color parameter b * (yellow-blue axis)

There was no significant effect of the treatment groups at baseline b*0 (*p* = 0.176) and after bleaching b*b (*p* = 0.463). The T-test for repeated measures revealed that the difference between b*0 and b*b was significant for all groups (*p* <0.001). These results can be observed in  Table 5.

**Table 5:**
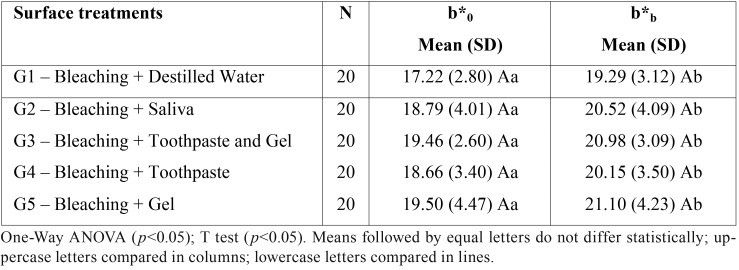
Means (standard deviation) of the values of the b*coordinate (blue-yellow axis) at baseline (b*0) and after bleaching (b*b) of the different surface treatments.

Two-way ANOVA showed that there was no significant effect on color change after the immersion in cola drink (b*s) between groups (*p* = 0.466), waiting times (*p* = 0.644) as well as their interaction (*p* = 0.984). The T-test for repeated measures showed that the difference between b*b and b*s in each group was statistically significant only for groups G3 (*p* = 0.001) and G4 (*p* = 0.018). The difference was not significant in groups G1 (*p* = 0.247), G2 (*p* = 0.362) and G5 (*p* = 0.099). These results are shown in  Table 6.

**Table 6:**
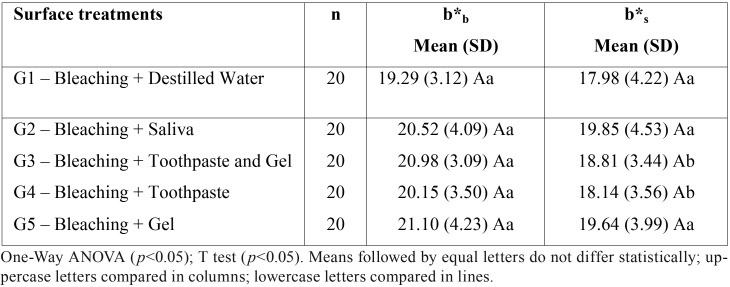
Means (standard deviation) of the values of the b* coordinate (blue-yellow axis) after bleaching (b*b) and after the contact with a cola drink (b*s) of the different surface treatments.

## Discussion

In the present study, the CIE L*a*b* system was used for color registration. The total difference of color (ΔE) is calculated from the differences between lightness and chroma coordinates (ΔL*, Δa*, Δb*). Considering the values of ΔEs, the surface treatments with calcium silicate, sodium phosphate and sodium monofluorophosphate and the waiting times for the contact with cola drink did not influence significantly the color maintenance of bleached enamel (*p*>0.05). The ΔEs>3 values confirm the clinically perceptible color change after pigmentation for all groups on both times evaluated.

Dentists instruct their patients to reduce the consumption of colouring beverages, especially those with low pH, during and after bleaching treatment since studies have reported that bleaching agents can change the enamel texture and morphology, favouring the reoccurrence of extrinsic coloration (12,3,8). However, previous studies have observed that the different times elapsed for the contact of the bleached enamel with chromophore beverages at 30 and 150 minutes, 24 hours, 7 days as well as immediately after the treatments do not significantly affect the bleaching results (6,14,4). These results are in agreement with the findings of the present study in which it was observed that the waiting times of 24 hours and 7 days for the contact with cola drink did not influence the pigmentation of bleached enamel.

Previous studies examined the effectiveness of a product based on calcium silicate, sodium phosphate and sodium monofluorophosphate and observed the deposition of minerals on the surface of eroded enamel (9,15,16,10). These studies evaluated the enamel after the surface treatments using scanning electron microscopy and superficial microhardness analysis. The susceptibility to pigmentation of the bleached enamel treated with this system was not previously performed. However, considering the mechanism of action of the fluoride associated with calcium silicate and sodium phosphate, some protective effect could be expected regarding color change, since it can be assumed that the surface alterations induced by the bleaching agent, such as surface porosity and permeability increase, would be minimized. In our study, no significant effect of the treatment groups was observed for any color parameter (L*, a* and b*) after immersion in cola drink, however, two-way ANOVA indicated a potential protective effect of the dual-phase serum associated with the toothpaste (b*) and the dual-phase serum used alone (L*, a* and b*) in maintaining the whitened color.

In the present study, in addition to the total color change, the color parameters of CIEL*a*b* system were also analyzed in baseline, after bleaching and after the surface treatments. The surface treatments had no effect before and after bleaching treatment for the parameters L* (lightness), a* (green-red axis) and b* (blue-yellow axis), demonstrating the uniformity of the sample used in this research. After bleaching, an increase in L* was observed for all surface treatments, demonstrating bleaching effectiveness. These findings are in accordance with the results presented in previous studies in which an increase in L* values was observed after bleaching (1,17,18)

Araújo *et al.* (2013) evaluated the mineral loss and color change of the enamel after bleaching and the pigmentation with cola, red wine and chocolate during one hour of immersion. Mineral loss was observed after bleaching and immersion in cola and red wine. Regarding enamel color change, there was no statistical difference between the groups before and after treatments, considering the L* coordinate (lightness) (19). Similar results were observed in the present study with reduction of lightness, although no significant statistical difference was observed in the comparison between the after bleaching and after pigmentation L * values.

Considering the parameter of color a* (green-red axis), previous studies observed a decrease in red chroma after bleaching (1,17,18) and an increase in its value after immersion in chromogenic beverages (20,12). In the present study, a significant effect of the artificial saliva and dual-phase serum on the red-green chromaticity parameter was observed, with an increase in a* value for the group treated with artificial saliva (G2) and a decrease of red chroma for the group treated with the dual-phase serum (G5). These results are in accordance with those observed for the parameter L* in which there was an increase of L* (lightness) for G5 and a decrease of its value for the other surface treatments. Such color coordinate variations (ΔL* and Δa*) suggest a protective effect of the dual-phase serum against bleached enamel pigmentation.

Considering the coordinate b* (blue-yellow axis), Guerrero *et al.* (2007) observed a decrease in the yellow chroma after bleaching (17). This *in vivo* study evaluated the color coordinates using 6.5% hydrogen peroxide daily for 3 weeks of home bleaching. Other *in vitro* studies that performed two to three sessions of in-office bleaching observed a decrease in the yellow chroma (12,1). This result, however, was not observed in the present study, that showed an increase in b* values in all groups after bleaching.

The analysis of the parameter b* after immersion in chromogenic beverages has shown an increase of the yellow axis (11,20). In our study, b* value decrease was observed after immersion in cola drink with a significant effect for groups G3 (toothpaste) and G4 (toothpaste and dual-phase serum). The reduction of yellow chroma in these groups suggests a toothpaste and serum protective effect on the maintenance of bleached color.

Analyzing the differences of the results found in similar studies, it is necessary to consider that the bleaching agent as well as the different treatment protocols used may have a great influence on the conclusions that were drawn, since its pH may interfere on enamel surface alteration and, consequently, on the color change observed (3,10). In addition, the substrate used in this research may have contributed to the variability of the results found. Although the similarities of bovine enamel with human enamel are demonstrated (21), the differences in chemical composition, morphology and physical properties between the two substrates should be considered in the interpretation of the results obtained from any experiment (22).

A high variability in the application protocol of RegenerateTM toothpaste was observed. The applications ranged from 7 days to four weeks (10,16) differing from this study in which the applications were made for only three consecutive days according to the manufacturer’s instructions, which may have also contributed to the different results.

The effects of surface remineralization with the system based on calcium silicate, sodium phosphate and fluoride have been demonstrated in *in vitro* and *in situ* studies, in which the deposition of minerals on the enamel exposed to an acid environment and its repair after demineralization were evaluated (9,10,23). The results of the present study suggest a protective effect of the dual-phase serum and toothpaste against bleached enamel pigmentation, probably due to the mineral reconstitution of the whitened tooth. New studies should be conducted to verify the protocols for the RegenerateTM system application and its effects on the morphological characteristics of bleached enamel, contributing to a better understanding of the mechanisms involved in the remineralization of the substrate.

## Conclusions

The surface treatments with the system based on calcium silicate, sodium phosphate and fluoride had a similar effect of artificial saliva and distilled water on the total color change of the enamel bleached with 35% hydrogen peroxide and stained in cola drink, but promoted a reduction of chromaticity (a*, b*) and an increase of lightness (L*), suggesting a potential protective effect against bleached enamel pigmentation.

The waiting times of 24 hours and 7 days for the contact with a cola drink were not relevant for the color change of the enamel bleached with 35% hydrogen peroxide.
